# Impact of different cover letter information and incentives on Veterans’ emotional responses to an unsolicited mailed survey about military traumas: a randomized, 3x2x2 factorial trial

**DOI:** 10.1186/s12874-022-01783-7

**Published:** 2022-12-01

**Authors:** Maureen Murdoch, Barbara A Clothier, Shannon Kehle-Forbes, Derek Vang, Siamak Noorbaloochi

**Affiliations:** 1 Section of General Internal Medicine, Minneapolis VA Health Care System, One Veterans Drive (111-0), Minneapolis, MN 55417 USA; 2 Center for Care Delivery and Outcomes Research, Minneapolis VA Health Care System, One Veterans Drive (152), Minneapolis, MN 55417 USA; 3 Department of Internal Medicine, University of Minnesota Medical School, 420 Delaware St SE, Minneapolis, MN 55455 USA; 4 National Center for, PTSD Women’s Health Sciences Division, VA Boston Healthcare System, 150 S Huntington Ave, Boston, MA 02130 USA; 5 Minneapolis Heart Institute Foundation, 920 E 28th St #100, Minneapolis, MN 55407 USA

**Keywords:** Randomized trial, Factorial design, Mailed survey, Combat, Military sexual trauma, Sexual assault, Ethics, Affect, Research subjects/psychology

## Abstract

**Background:**

Altering cover letter information to reduce non-response bias in trauma research could inadvertently leave survey participants unprepared for potentially upsetting questions. In an unsolicited, mailed survey, we assessed participants’ change in affect post-survey after altering key cover letter information and promising different incentives. We tested direct and indirect effects of participants carefully reading the cover letter on changes in their affect post-survey.

**Methods:**

In a 3X2X2 randomized, factorial trial, 480 male and 480 female, nationally representative Veterans who were applying for posttraumatic stress disorder disability benefits were randomized to receive one of 12 different cover letters. The cover letters provided general versus more explicit information about the survey’s trauma content and how their names were selected for study; we also promised different incentives for returning the survey. The main outcome was change in affect post-survey. We examined five potential moderators: combat or military sexual trauma exposure, posttraumatic stress disorder or serious mental illness diagnosis, and recency of military service. Mediators between reading the cover letter carefully and post-survey affect included how participants rated the cover letters’ information and whether they thought the cover letters prepared them for the survey’s content. A Bonferroni corrected alpha of 0.003 was the threshold for statistical significance.

**Results:**

One hundred ninety men and 193 women reported their pre-and post-survey affect. Across all study conditions, out of 16 possible points, the net change in affect post-survey was less than a quarter-point for men and women. Mean changes in post-survey affect did not differ statistically significantly across any of the study factors (*p*s > 0.06); nor were there statistically significant interactions between any of the study factors and the 5 moderators after accounting for multiple comparisons (*p*s > 0.02). After controlling for pre-survey affect, reading the cover letter carefully had small effects on changes in post-survey affect, with larger associations seen in the women compared to men. Mediators’ effects were often in opposite directions for men and women.

**Conclusion:**

General descriptions of a survey’s trauma content appear ethically defensible. Research on cover letters’ impacts on survey participants’ emotional reactions and how those impacts differ by gender is needed.

**Supplementary Information:**

The online version contains supplementary material available at 10.1186/s12874-022-01783-7.

## Background

Self-report surveys have informed much of our understanding of trauma’s epidemiology in military samples, e.g., [1–4]. Unfortunately, negative emotional reactions induced by trauma-related queries could cause some trauma survivors to systematically opt out of responding. This, in turn, could lead to skewed samples and biased conclusions. For example, male Veterans with histories of military sexual trauma are substantially less likely to participate in self-report surveys that cover such topics compared to their counterparts without sexual trauma history [5]. Their under representation among survey respondents could cause investigators to underestimate the burden of sexual assault in men and to miss causal etiologies suitable for intervention. Reducing non-response bias therefore represents a social good that could better our understanding of trauma epidemiology and improve trauma interventions, regardless of the stressor under investigation.

For postal surveys, potential participants typically learn what topics will be addressed from an accompanying cover letter. Depending on how and what information is provided, cover letters could serve as vehicles for inducing or reducing non-response bias. Emphasizing a postal survey’s combat content, for example, preferentially induces combat-exposed Veterans to participate [6]. Not mentioning combat content at all might therefore lead to more representative participation from Veterans without combat exposure. The societal good gained from reduced non-response bias must be balanced against risks to individual survey participants, however. Failing to disclose survey content that might remind participants of prior traumas could trigger trauma-related symptoms or upset them emotionally.

In a postal survey related to trauma, we tested whether altering cover letter information would induce or reduce non-response bias in a sample of US Veterans applying for posttraumatic stress disorder (PTSD) disability benefits [6]. In the present study, we examine whether those cover letter alterations impacted participants’ emotional reactions. A priori, we anticipated that warning potential participants that their survey asked about specific traumas would help them better prepare for and tolerate trauma-related items. We anticipated that cover letter warnings would be most helpful to those with the relevant trauma history –that is, telling sexual assault survivors that the survey asked about unwanted sexual attention would be more effective in reducing post-survey upset than telling them the survey asked about combat. Other personal vulnerability factors that we thought might moderate associations between cover letter information and post-survey upset included whether the participant had a diagnosis of PTSD or of other serious mental illness [7–10]. We also speculated that participants more recently separated from military service might experience more post-survey upset compared to those with more distant military service. Most prior research examining Veterans’ emotional reactions to military trauma-related surveys have included samples who separated from military service 2 to 3 decades earlier [7, 8, 10]. Having had several years in which to process or learn to cope with trauma reminders, such survey recipients might not react as strongly to unexpected trauma reminders compared to individuals with more recent experiences

Since the cover letter carried the stimulus, we anticipated that individuals who did not read the cover letter or merely skimmed it would be less prepared for the survey’s content—and thus more upset post-survey—compared to those who read the cover letter carefully. We likewise anticipated that participants who, having read the cover letter, believed that the cover letter prepared them well for the survey’s content or gave them enough information about the survey’s content would experience less post-survey upset compared to participants who believed the cover letter did not prepare them well or give enough information.

Our original non-response bias study also varied the information provided potential participants about how we obtained their name for study and how much incentive we would pay for a returned survey. Although we did not have specific hypotheses tied to these study arms in terms of emotional reactions, any decisions to implement similar strategies in future research would require understanding their emotional impacts. Therefore, outcomes are reported for these two factors as well.

## Methods

### Study Design and Human Studies Oversight

The study is based on a gender-blocked, randomized, 3X2X2 factorial comparison trial. The Minneapolis VA Health Care System’s Internal Review Board for Human Studies reviewed and approved the study protocol (#4495-B). All analyses were pre-planned. Data were collected between February and August 2016.

### Population and Setting

The population was 17,615 Operation Enduring Freedom, Operation Iraqi Freedom, or Operation New Dawn (OEF/OIF/OND) veterans who had pending claims for Department of Veterans Affairs (VA) PTSD disability benefits between March 1, 2015 and November 14, 2015. From this frame, we randomly selected 480 men and 480 women for survey. As reported elsewhere [6], median age was 33.0 years (mean = 35.2, SD = 8.9, range = 19-67); 58.1% of the men and 40.6% of the women were white, 19.6% of the men and 36.5% of women were black or African American, and 13.3% of men and 10.2% of women were Hispanic. Almost a third of the sample (32.4%) had left active service within two years of the survey, and 59.3%, within 5 years (mean years since separation = 5.1, SD= 4.0, range = 0-14). Among the men, 67.1% carried administrative flags for combat exposure and 2.1% had screened positive for military sexual assault or severe, physical sexual harassment. Among women, 41.3% carried administrative flags for combat exposure and 45.6% had screened positive for military sexual assault or severe, physical sexual harassment. 71.6% of men and 61.1% of women had a chart diagnosis of PTSD; 7.1% of men and 10.3% of women had been diagnosed with bipolar disorder, schizophrenia, or schizoaffective disorder.

### Study arms

Veterans were randomized to receive 1 of 12 possible cover letters, depending on the factorial combination they were assigned to. The first factor, of greatest interest here, altered the information provided in the cover letter about the survey’s topics in one of three ways: potential participants were told the survey asked about “combat,” about “unwanted sexual experiences in the military,” or about “lifetime and military experiences that can affect well-being.” The second factor altered what Veterans were told about their selection for the study: Veterans were told that they had been randomly selected for inclusion from “a Department of Veterans Affairs list of Veterans who filed a disability claim” or from “a Department of Veterans Affairs list of Veterans who served during OEF/OIF/OND.” The last factor randomized participants to receive $20 or $40 after returning a completed questionnaire. Participants were told which incentive they would receive in the cover letter. Except for the 3 varied factors, cover letters were exactly the same in appearance, length, text, formatting, and content.

### Protocol

All potential participants received a pre-notification letter, which alerted recipients of the coming survey but did not describe its content. One week later, we mailed the cover letter and a 22-page questionnaire to Veterans’ homes. In addition to asking about combat and military sexual trauma—the presumed trauma reminders—the questionnaire asked about physical and social functioning, mental health symptoms, and pain. At two-week intervals, non-respondents were mailed a post-card reminder followed by two more copies of the questionnaire. The last questionnaire was mailed via United Parcel Service’s 3-day delivery service.

In all cover letters, Veterans were explicitly told that they might find some questions personal or upsetting, and they were specifically told that it was “ok” to skip any upsetting questions. We also provided all Veterans with several copies of help-line phone numbers in their mailing packets, throughout the questionnaires’ pages, and on the questionnaire’s backpiece. The questionnaire’s frontispiece included a prominent warning that some items might feel personal or sensitive to the Veteran and informed them that they could skip any questions they didn’t wish to answer.

### Study outcome

The main outcome was Veterans’ emotional reactions to the survey, operationalized as their affective change in valence (happiness/sadness) and arousal (calmness/tenseness) post-survey compared to pre-survey.

### Moderators and mediators

Hypothesized moderators included Veterans’ military trauma exposures (combat or military sexual trauma), mental health diagnoses (PTSD or serious mental illness), and how recently they separated from the armed forces. Mediators included how carefully participants read the cover letter, if at all; how well they believed the cover letter prepared them for the survey’s content; and whether they believed the cover letter gave them enough information about the survey’s content. The second mediator, how well respondents thought the cover letter prepared them for the survey’s content, was intended to assess the cover letter’s global impact—not only the specific content information they had been provided, but also the warning given to everyone that some questions might be upsetting, that they could skip questions, that participation was voluntary, and so on. The third mediator, whether respondents had been given enough information about the types of questions they would find, was intended to assess the study stimulus itself, namely, what respondents had been told about the survey’s topic content.

### Measures

#### Main outcome

 We used the *Self-Assessment Manikin* (SAM) [11] to assess each participant’s valence and arousal immediately before and after completing the questionnaire. For valence, a continuum of 5 stylized human figures or manikins depicts feeling “very happy” to “very sad.” A similar continuum of 5 stylized manikins depicts feeling “very calm” to “very tense” for arousal. Intermediate response options allow participants to choose a feeling halfway between two manikins, resulting in nine total response options for both valence and arousal. Thus, valence and arousal scores may range from 1 to 9, and a score of 5 represents the mid-point.

The questionnaire’s first full page presented the valence and arousal manikins and asked participants to select “how happy or sad (or tense or calm) are you *right now*?” The same manikins were repeated on the last full page of the questionnaire, and participants were again asked to mark how happy or sad (or tense or calm) they felt “*right now*.” Score changes, which may range from -8.0 to 8.0, were calculated by subtracting post-survey SAMs from participants’ pre-survey scores. Positive score changes indicate more sadness or tenseness post-survey; negative score changes, less sadness or tenseness post-survey. A one-unit change on either SAM scale indicates a change halfway between manikins; a two-unit change reflects movement from one full manikin to the next. Clinically important score changes have not been established for the SAM. However, 1.2-point score changes have been reported as the mean affective difference between reading print advertisements with and without background music [12], and 1.7 to 2.3 points, the mean affective difference before and after participating in an exercise class [13].

#### Moderators

 We abstracted Veterans’ combat and military sexual trauma exposures from the Veterans Benefits Administration corporate databases and from the VA’s Informatics and Computing Infrastructure (VINCI) Corporate Data Warehouse. We also abstracted whether participants had received any diagnosis of PTSD in the 180 days prior to the first mail date (February 1, 2016) or received a PTSD disability award. If yes, they were considered to have PTSD. We operationalized serious mental illness as having been diagnosed with bipolar disorder, schizophrenia, or schizoaffective disorder within the 180 days prior to the first mail date and abstracted this information from VINCI. To determine the time elapsed since participants left the armed forces, we obtained their release from active duty date from VBA corporate databases and subtracted that result from the date we mailed their first survey. We trichotomized results into 0-5 years, 6-10 years, and > 10 years.

#### Mediators

Near the end of the questionnaire, just prior to the second SAM, we asked respondents to rate “how carefully did you read the cover letter that came with this survey” on a scale of 0 (“Didn’t read”) to 4 (“Very carefully”); to rate “how much information did the cover letter give you regarding the kinds of questions you would find in the survey” on a scale of 0 (“Doesn’t apply, didn’t read”) to 3 (“The cover letter gave me too much information”); and to rate “how well did the cover letter prepare you for the types of questions you found in the survey” on a scale of 0 (“Doesn’t apply, didn’t read”) to 4 (“I was very prepared”).

### Power

Analysis was based on the number of survey respondents. No formal power analysis was done.

### Analysis

Men’s and women’s results are reported separately to account for our stratified sampling strategy. Of the 199 men and 211 women who returned at least partially completed surveys, we limited analyses to the 190 men and 193 women who completed both pre- and post-survey SAMs. For each gender, we report mean pre-survey affect and change in affect overall. We also report the change in affect by each of the study’s 3 factorial assignments and by each of the hypothesized moderators. We report response frequencies for the 3 hypothesized cover letter mediators overall and by each of the 3 factorial assignments. We used Pearson χ^2^tests to assess for dependent relationships between moderators and mediators across factorial assignments and ANOVA to compare post-survey changes in valence and arousal across factorial assignments and moderators. We used ANCOVA to examine potential interactions between moderators and factorial assignments on post-survey changes in valence and arousal while controlling for Veterans’ pre-survey affect. We present the latter results using box plots. We used structural equation modeling (SAS CALIS procedure, version 9.4) to assess the direct and indirect effects of reading the cover letter carefully on post-survey affect while controlling for pre-survey affect. We used a Bonferroni-corrected alpha of 0.003 as the threshold for statistical significance.

## Results

The Supplementary Table (Additional file) shows the distribution of military exposures, PTSD, serious mental illness diagnosis, and years since leaving the armed forces across each of the study’s factors after stratifying by gender. None of the distributions differed significantly by what Veterans had been told about the survey’s content or about how they had been selected for participation. Although not meeting criterion for statistical significance, there were more women participants who had separated from the military within 0-5 years and fewer who had separated more than 10 years ago among those offered the $20 incentive compared to women offered the $40 incentive (*p* = 0.01). Only 4 men with military sexual trauma flags participated in the study. By chance, none were randomized to receive the cover letter stating the survey asked about unwanted sexual attention.

### Main Factor Effects

As Table 1 shows, participants’ overall net, pre-survey affect was approximately mid-point for valence and arousal. Post-survey, all net score changes were less than 1, or less than the half-way point between manikins. Men were, on net, 0.25 (SD=1.25) points sadder and 0.14 (SD= 1.41) points tenser post-survey compared to pre-survey; on net, women were 0.22 (SD=1.40) points sadder and 0.20 points tenser (SD=1.48) post-survey. The average post-survey change in Veterans’ affect did not differ statistically significantly by what they were told about the survey’s content (*p*s > 0.12). Veterans’ change in affect post-survey also did not differ statistically significantly by what they were told about their selection process (*p*s > 0.06) or by what incentive they were promised (*p*s > 0.12).

**Table 1:** Pre-survey affect and change in affect overall; change in affect post-survey by study factors

Affect	Affect Overall		Topics Covered by Survey			How Name was Obtained		Incentive	
	Pre-Survey	Change after Survey	Combat	Military Sexual Trauma	Life Experiences that Affect Well-Being	List of OEF/OIF/OND Veterans	List of Veterans applying for Disability Benefits	$20	$40
Men	*N* = 190	*N* = 190	*n* = 68	*n* = 66	*n* = 56	*n* =84	*n* =106	*n* =81	*n* =109
Valence	5.01 (1.68)	0.25 (1.25)	0.46 (1.27)	0.02 (0.97)	0.27 (1.47)	0.11 (1.10)	0.36 (1.35)	0.09 (1.31)	0.37 (1.20)
Arousal	5.07 (1.94)	0.14 (1.42)	0.13 (1.28)	0.17 (1.17)	0.11 (1.81)	0.12 (1.35)	0.15 (1.47)	0.11 (1.47)	0.16 (1.38)
Women	*N* = 193	*N* = 193	*n* = 59	*n* = 67	*n* = 67	*n* =92	*n* =101	*n* =87	*n* =106
Valence	5.40 (1.81)	0.22 (1.40)	0.31 (1.36)	0.15 (1.34)	0.21 (1.49)	0.08 (1.26)	0.35 (1.50)	0.34 (1.44)	0.11 (1.35)
Arousal	5.27 (1.89)	0.20 (1.48)	0.41 (1.63)	-0.03 (1.38)	0.25 (1.42)	-0.01 (1.35)	0.40 (1.57)	0.25 (1.60)	0.16 (1.37)

Results are reported as means with standard deviations in (). Positive score changes signify more sadness (valence) or tenseness (arousal) post-survey compared to pre-survey. Negative score changes signify less sadness (valence) or tenseness (arousal) post-survey compared to pre-survey. Score changes’ possibilities range from -8 to 8.

OEF/OIF/OND = Operation Enduring Freedom, Operation Iraqi Freedom, Operation New Dawn.

### Moderating effects

**Fig. 1:**
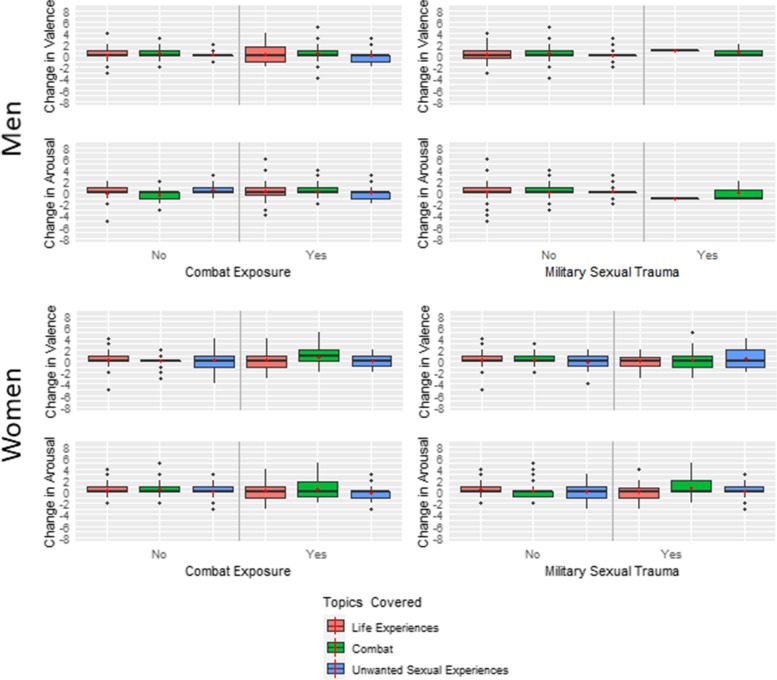
Post-Survey Change in Affect by Participants’ Military Trauma Exposures and What They were Told about the Survey’s Content. Box plots of participants’ post-survey change in affect according to their military trauma history and what they were told about the survey’s content. Men’s results are shown in the top 4 panels, and women’s, in the bottom 4. Red dots indicate the mean change and black bars, the median change. Positive numbers indicate more sadness or tenseness post survey compared to pre-survey; negative numbers, less sadness or tenseness. No men with a military sexual trauma history were assigned to the cover letter indicating that the survey asked about unwanted sexual attention while in the military.

Although we had anticipated interactions between what participants had been told about the survey’s content, their military trauma exposures, and their change in affect post-survey, the box plots in Figure 1 show that this was not the case. Regardless of what participants were told about the survey’s topics or their exposures to combat or military sexual trauma, average post-survey changes in valence and arousal were less than or equal to one point (all *p*s > 0.07). Supplementary Figure 1 (additional file) shows that there were likewise no interactions between what participants had been told about the survey’s topics and having a PTSD or serious mental illness diagnosis or being more recently separated from the armed forces on changes in post-survey affect (all *p*s > 0.05).

The remaining boxplots in Supplementary Figures 2 – 5 show the interactions between what Veterans were told about their selection process or the incentive they were promised, their military exposures, PTSD or serious mental illness diagnoses, and when they separated from the armed forces. None of these tests for moderation met criterion for statistical significance. For all contrasts across all the groups, net changes in affect post-survey were 1 point or less.

### Mediating effects

Table 2 shows that, overall, 2.6% of men and 4.7% of women said they did not read the cover letter, while 80.1% of men and 73.4% of women said they read the cover letter “somewhat” to “very” carefully. On subsequent questions, 6.8% of men and 7.9% - 9.5% women said they didn’t read the cover letter. Overall, 77.4% of men and 77.8% of women said the cover letter gave them “just enough” information about the types of questions they found in the survey, and 61.6% of men said the survey prepared them for the types of questions they found in the survey “somewhat” to “very” well, as did 69.3% of women. The care with which participants read the cover letter and their ratings of how much information they obtained from the cover letter or how well the cover letter prepared them for the survey’s questions were similar across all the study’s factors (*p*s > 0.07).

**Table 2:** Cover letter mediators overall and by study factors

Cover Letter Mediator	Overall	Topics Covered by Survey			How Name was Obtained		Incentive	
		Combat	Military Sexual Trauma	Life Experiences that Affect Well-Being	List of OEF/OIF/OND Veterans	List of Veterans applying for Disability Benefits	$20	$40
Read cover letter carefully								
Men	*N* = 190	*n* = 68	*n* = 66	*n* = 56	*n* =84	*n* =106	*n* =81	*n* =109
Not at all	5.8	4.4	7.6	5.4	8.3	3.8	4.9	6.4
A little bit	11.6	5.9	15.2	14.3	11.9	11.3	9.9	12.8
Somewhat	41.1	47.1	28.8	48.2	40.5	41.5	46.9	36.7
Very	39.0	39.7	45.5	30.4	35.7	41.5	33.3	43.1
Didn’t read	2.6	2.9	3.0	1.8	3.6	1.9	4.9	0.9
Women	*n* = 193	*n* = 59	*n* = 67	*n* = 67	*n* =92	*n* =101	*n* =87	*n* =106
Not at all	9.4	10.3	9.0	9.0	13.2	5.9	9.3	9.4
A little bit	12.5	15.5	9.0	13.4	8.9	15.8	12.8	12.3
Somewhat	32.8	37.9	29.9	31.3	31.9	33.7	33.7	32.1
Very	40.6	31.0	47.8	41.8	42.9	38.6	38.4	42.5
Didn’t read	4.7	5.2	4.5	4.5	3.3	5.9	5.8	3.8
Information provided								
Men	*N* = 190	*n* = 68	*n* = 66	*n* = 56	*n* =84	*n* =106	*n* =81	*n* =109
Not enough	13.2	13.2	9.1	17.9	14.3	12.3	12.4	13.8
Just enough	77.4	76.5	78.8	76.8	70.2	83.0	76.5	78.0
Too much	2.6	2.9	4.6	0.0	4.8	0.9	1.2	3.7
Didn’t read	6.8	7.4	7.6	5.4	10.7	3.8	9.9	4.6
Women	*N* = 193	*n* = 59	*n* = 67	*n* = 67	*n* =92	*n* =101	*n* =87	*n* =106
Not enough	11.6	10.5	9.0	15.4	11.2	12.0	7.1	15.4
Just enough	77.8	79.0	79.1	75.4	77.5	78.0	81.2	75.0
Too much	1.1	0.0	3.0	0.0	1.1	1.0	1.2	1.0
Didn’t read	9.5	10.5	9.0	9.2	10.1	9.0	10.6	8.7
Prepared respondent for questions in the survey								
Men	*N* = 190	*n* = 68	*n* = 66	*n* = 56	*n* =84	*n* =106	*n* =81	*n* =109
Not at all	5.8	5.9	4.6	7.1	7.1	4.7	7.4	4.6
A little bit	25.8	20.6	25.8	32.1	25.0	26.4	24.7	26.6
Somewhat	30.5	41.2	27.3	21.4	30.0	31.1	33.3	28.4
Very	31.1	26.5	34.9	32.1	25.6	33.0	24.7	35.8
Didn’t read	6.8	5.9	7.6	7.1	9.5	4.7	9.9	4.6
Women	*N* = 193	*n* = 59	*n* = 67	*n* = 67	*n* =92	*n* =101	*n* =87	*n* =106
Not at all	5.8	6.9	4.5	6.3	5.6	6.1	3.5	7.8
A little bit	16.9	20.7	13.4	17.2	14.4	19.2	11.6	21.4
Somewhat	37.0	41.4	32.8	37.5	38.9	35.4	39.5	35.0
Very	32.3	22.4	40.3	32.8	33.3	31.3	36.1	29.1
Didn’t read	7.9	8.6	9.0	6.3	7.8	8.1	9.3	6.8

Results are reported as column percentages. Column percentages may not add to 100% secondary to rounding.

OEF/OIF/OND = Operation Enduring Freedom, Operation Iraqi Freedom, Operation New Dawn.

**Fig. 2:**
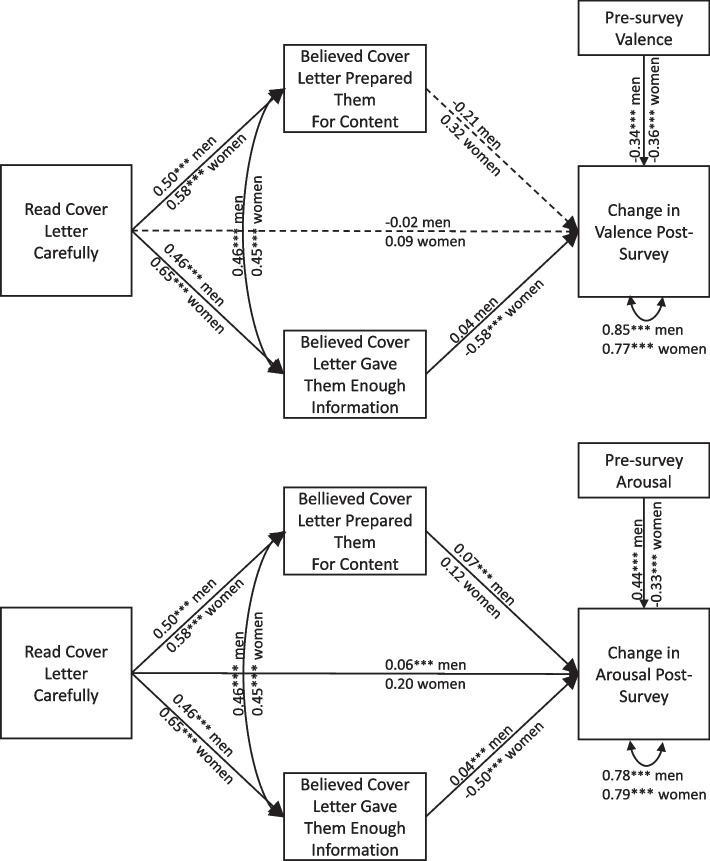
Mediation between How Carefully Participants Read the Cover Letter and Change in Affect Post-Survey, Controlling for Pre-Survey Affect. Results of a structural equation model with standardized β coefficients. Men’s β coefficients are shown on top, and women’s, directly underneath. Dashed line indicates statistically non-significant association. ***p < 0.001

Even though participants were not significantly more or less likely to read the cover letter carefully based on which version they received, Figure 2 shows that reading the cover letter more carefully was positively associated with participants believing the cover letter prepared them for the survey’s questions and with participants rating the cover letter information more favorably (*p*s < 0.001). After controlling for pre-survey affect, there were no direct associations between reading the cover letter more carefully and post-survey changes in valence (*p*s > 0.26). There was a small, direct, positive association between reading the cover letter more carefully and reporting increased arousal post-survey for men (*p* < 0.001). Direct associations between feeling prepared by the cover letter or feeling one had received enough information from the cover letter and post-survey changes in affect differed by gender. For example, believing the cover letter provided enough information about the survey was negatively associated with post-survey changes in women’s valence and arousal (*p*s< 0.001), suggesting greater happiness and calmness post-survey. In men, believing the cover letter provided enough information about the survey was not associated with men’s changes in valence (*p* = 0.65) and predicted greater arousal post-survey compared to pre-survey (*p* < 0.001).

## Discussion

The present study showed that most participants responding to this trauma-related survey had very small net changes in their affect compared to pre-survey. Contrary to expectations, this change was not influenced by what participants had been told about the survey’s content. Likewise, there were no moderating effects between what participants were told about the survey’s content and the change in their post-survey affect based on military trauma exposures, PTSD or serious mental illness diagnoses, or how recently they had left active duty. Reassuringly, most participants read the cover letter at least “somewhat carefully.” However, there were no direct associations between reading the cover letter carefully and post-survey changes in valence. Counter to expectations, reading the cover letter more carefully was associated with larger increases in men’s arousal post-survey; however, while statistically significant, the effect size was small.

Cover letters carry dual purposes in postal surveys. First, they are used to generate enthusiasm about the research and encourage participation. Second, they convey required elements of informed consent, including the risks and benefits of participating in the research. While substantial research has assessed the impact of various cover letter elements on response rates [14], there are very few systematic assessments of how well cover letters inform participants about a survey’s risks and benefits. Lykes and Meyers [15] found that just 36% of respondents to a push-to-web survey about car ownership said they had read and understood the cover letter before completing their survey. In a series of cognitive interviews for the American Community Survey, a US Census supplement, Martinez, et al. [16] showed that only 60% of participants noticed that cover letter information continued onto a back page; of those noticing, only 13% read the back text carefully. Forty percent of participants did not see the cover letter statement indicating participation was required by law. In the present study, 2.6% to 6.8% of men and 4.7% to 7.9% of women said they did not read the cover letter. This low rate of non-reading suggests that the present population was more inclined toward systematic and deliberative decision-making approaches to survey participation [17] compared to the Lykes and Meyers and Martinez, et al. samples. We find it reassuring that the different cover letter versions did not importantly impact how carefully participants read their letters, rated their letters’ adequacy of information, or prepared them for the survey’s content. Individuals belonging to groups that we might anticipate as being at greater risk for post-survey upset, such as those with PTSD or relevant traumas, reported relatively small post-survey changes in affect, and this did not differ according to the cover letter version they received. These results suggest that cover letters describing a survey’s trauma content in more general terms, e.g., “lifetime and military experiences that can affect well-being,” do not carry higher risks or represent suboptimal information compared to more explicit descriptions.

Although incidental to our main hypothesis, our results also suggest that providing less explicit information about how Veterans’ names were selected or offering a higher or lower incentive did not adversely affect their emotional reactions to the survey. Concerns have sometimes been raised that higher incentives might cause study participants—especially vulnerable study participants—to discount the risks that research poses [18]. However, our data suggest that study participants offered $40 incentives read the cover letter as carefully as those offered $20. Veterans’ other ratings of the cover letter were also similar, regardless of what we told them about their name’s selection or the incentive we promised.

Contrary to expectations, we found no-to-small direct associations between reading the cover letter more carefully and changes in participants’ post-survey affect. For men’s post-survey arousal, the direction of effect was opposite expectations. Most of the effect of reading the cover letter carefully on post-survey affect was indirect and mediated through participant’s believing that the cover letter prepared them for the survey’s content or gave them enough information about the survey’s content. On net, these associations were larger for the women than for the men. Compared to other women and as expected, women who believed the cover letter provided enough information about the survey’s content were happier and calmer post-survey compared to pre-survey. However, there was no statistically significant association between women believing the cover letter prepared them for the survey’s content and change in their valence or arousal post-survey. Compared to other men, those who believed the cover letter prepared them for the survey’s content were more aroused post-survey compared to pre-survey. Future research should plumb these discrepancies and explore strategies by which cover letters can better prepare participants for potentially upsetting survey content. Understanding how and why men and women react to cover letters differently would also help optimize cover letters’ content.

Results speak only to this group of participants and do not apply to survey recipients who either opted out of the survey or failed to fully complete the survey. Generalizability to other Veterans, to non-Veterans, or to individuals who have experienced other types of trauma are unknown. To the extent that we surveyed a highly traumatized population who might be particularly likely to be emotionally upset by an unsolicited, trauma-related survey, our findings of small net changes in affect are reassuring. Our study focused on whether changing cover letter information would impact participants’ impressions of the cover letter, but not the underlying mechanisms of how those impressions were formed. People with more inherent emotional resilience may be more inclined to read cover letters thoroughly or to rate them more positively compared to those with lower emotional resiliency. Thus, the cause-and-effect association between participants’ cover letter ratings and post-survey changes in affect are unclear. Veterans may have multiple traumatic experiences (e.g. combat plus military sexual trauma), so specifically mentioning only one of the survey’s trauma topics could have left them unprepared for the other topic. While this could have dampened the cover letter’s impact, such individuals should have been randomly distributed across the intervention arms, resulting in unbiased effect size estimates.

Our earlier analysis of this group showed that cover letters explicitly identifying combat as a survey topic resulted in over participation of men and women with combat exposure by approximately 4.5 percentage points, while explicitly identifying unwanted sexual attention as a survey topic resulted in over participation by women with military sexual trauma by almost 3 percentage points. Men with exposure to military sexual trauma under participated regardless of the cover letter information provided [6]. The clinical importance of these selection biases is unclear, and none were statistically significant. The present analysis extends these findings by showing that altering the cover letter information or offering different incentives did not cause undue emotional upset in the participants, even among those with combat or military sexual trauma exposure, with PTSD or serious mental illness, or with more recent military separation. To the extent that trauma researchers might wish to avoid selection biases on the order of 3% to 5%, using more general descriptions of a survey’s trauma content appears ethically defensible. More research on cover letters’ impact on survey participants’ emotional reactions to unsolicited, mailed surveys and how those impacts might differ by gender is needed.

## Supplementary Information

**Additional file 1: Supplementary Table. **Characteristics of Participants Overall and by Study Factors

**Additional file 2: Supplementary Figure 1. **“Post-Survey Change in Affect by Participants’ Other Vulnerability Factors and What They were Told about the Survey’s Content.” Box plots of participants’ post-survey change in affect according to their other vulnerability factors and what they were told about the survey’s content. Men’s results are shown in the top 4 panels, and women’s, in the bottom 4. Red dots indicate the mean change and black bars, the median change. Positive numbers indicate more sadness or tenseness post survey compared to pre-survey; negative numbers, less sadness or tenseness

**Additional file 3: Supplementary Figure 2**. “Post-Survey Change in Affect by Participants’ Military Trauma Exposures and How their Name was Obtained.” Box plots of participants’ post-survey change in affect according to their military trauma history and what they were told about how their name was obtained for inclusion in the study. Men’s results are shown in the top 4 panels, and women’s, in the bottom 4. Red dots indicate the mean change and black bars, the median change. Positive numbers indicate more sadness or tenseness post survey compared to pre-survey; negative numbers, less sadness or tenseness.

**Additional file file 4: Supplementary Figure 3.** “Post-Survey Change in Affect by Participants’ Military Trauma Exposures and the Incentive They were Promised.” Box plots of participants’ post-survey change in affect according to their military trauma history and the incentive they were promised. Men’s results are shown in the top 4 panels, and women’s, in the bottom 4. Red dots indicate the mean change and black bars, the median change. Positive numbers indicate more sadness or tenseness post survey compared to pre-survey; negative numbers, less sadness or tenseness.

**Additional file 5: Supplementary Figure 4.** “Post-Survey Change in Affect by Participants’ Other Vulnerability Factors and How their Name was Obtained.” Box plots of participants’ post-survey change in affect according to their other vulnerability factors and what they were told about how their name was obtained for inclusion in the study. Men’s results are shown in the top 4 panels, and women’s, in the bottom 4. Red dots indicate the mean change and black bars, the median change. Positive numbers indicate more sadness or tenseness post survey compared to pre-survey; negative numbers, less sadness or tenseness.

**Additional file 6: Supplementary Figure 5.** “Post-Survey Change in Affect by Participants’ Other Vulnerability Factors and the Incentive They were Promised.” Box plots of participants’ post-survey change in affect according to their other vulnerability factors and the incentive they were promised. Men’s results are shown in the top 4 panels, and women’s, in the bottom 4. Red dots indicate the mean change and black bars, the median change. Positive numbers indicate more sadness or tenseness post survey compared to pre-survey; negative numbers, less sadness or tenseness.

## Data Availability

The data that support the findings of this study are available from the corresponding author (MM) upon reasonable request and with local Minneapolis VA Health Care System IRB permission.

## Abbreviations

ANCOVA: Analysis of covariance
ANOVA: Analysis of variance
CALIS: Covariance analysis of linear structural equations
OEF/OIF/OND: Operations Enduring Freedom, Iraqi Freedom, or New Dawn
PTSD: Posttraumatic stress disorder
SAS: Statistical Analysis System
VA: Department of Veterans Affairs
VINCI: Department of Veterans Affairs Informatics and Computing Infrastructure
