# Supplementary material for: Impact of different cover letter information and incentives on Veterans’ emotional responses to an unsolicited mailed survey about military traumas: a randomized, 3x2x2 factorial trial
Source: BMC Med Res Methodol. 2022 Dec 1;22:308. doi: 10.1186/s12874-022-01783-7 (PMC9714177; doi:10.1186/s12874-022-01783-7)
Supplement: Supplementary file 1 — Additional file 1: Supplementary Table. Characteristics of Participants Overall and by Study Factors [file 12874_2022_1783_MOESM1_ESM.docx]

**Supplementary Table. Characteristics of Participants Overall and by Study Factors.**

| Characteristic | Overall | Topics Covered by Survey | | | How Name was Obtained | | Incentive | |
| --- | --- | --- | --- | --- | --- | --- | --- | --- |
|  |  | Combat | Military Sexual Trauma | Life Experiences that Affect Well-Being | List of OEF/OIF/OND Veterans | List of Veterans applying for Disability Benefits | $20 | $40 |
| Men | N = 190 | n = 68 | n = 66 | n = 56 | n =84 | n =106 | n =81 | n =109 |
| Combat exposure | 68.4 | 70.6 | 71.2 | 62.5 | 69.0 | 67.9 | 63.0 | 72.5 |
| Military sexual trauma | 2.1 | 4.4 | 0.0 | 1.8 | 1.2 | 2.8 | 2.5 | 1.8 |
| PTSD diagnosis | 71.5 | 72.1 | 74.2 | 67.9 | 75.0 | 68.9 | 72.8 | 70.6 |
| Serious mental illness | 6.8 | 4.4 | 4.5 | 12.5 | 9.5 | 4.7 | 6.2 | 7.3 |
| Separated from military |  |  |  |  |  |  |  |  |
| 0-5 years ago | 60.0 | 64.7 | 57.6 | 57.1 | 58.3 | 61.3 | 56.8 | 62.4 |
| 6-10 years ago | 31.1 | 27.9 | 33.3 | 32.1 | 31.0 | 31.1 | 30.9 | 31.2 |
| >10 years ago | 8.9 | 7.4 | 9.1 | 10.7 | 10.7 | 7.5 | 12.3 | 6.4 |
| Women | N = 193 | n = 59 | n = 67 | n = 67 | n =92 | n =101 | n =87 | n =106 |
| Combat exposure | 46.1 | 44.1 | 38.8 | 55.2 | 41.3 | 50.5 | 47.1 | 45.3 |
| Military sexual trauma | 43.5 | 42.4 | 49.3 | 38.8 | 40.2 | 46.5 | 43.7 | 43.4 |
| PTSD diagnosis | 61.1 | 55.9 | 59.7 | 67.2 | 59.8 | 62.4 | 59.8 | 62.3 |
| Serious mental illness | 9.8 | 8.5 | 13.4 | 7.5 | 9.8 | 9.9 | 11.5 | 8.5 |
| Separated from military |  |  |  |  |  |  |  |  |
| 0-5 years ago | 59.1 | 61.0 | 55.2 | 61.2 | 63.0 | 55.4 | 66.7 | 52.8 |
| 6-10 years ago | 26.9 | 40.2 | 23.9 | 32.8 | 26.1 | 27.7 | 27.6 | 26.4 |
| >10 years ago | 14.0 | 15.3 | 20.9 | 6.0 | 10.9 | 16.8 | 5.7 | 20.8 |

Results are reported as column percentages.

OEF/OIF/OND = Operation Enduring Freedom, Operation Iraqi Freedom, Operation New Dawn. PTSD = Posttraumatic stress disorder
