# Supplementary material for: Impact of different cover letter information and incentives on Veterans’ emotional responses to an unsolicited mailed survey about military traumas: a randomized, 3x2x2 factorial trial
Source: BMC Med Res Methodol. 2022 Dec 1;22:308. doi: 10.1186/s12874-022-01783-7 (PMC9714177; doi:10.1186/s12874-022-01783-7)
Supplement: Supplementary file 2 — Additional file 2: Supplementary Figure 1. “Post-Survey Change in Affect by Participants’ Other Vulnerability Factors and What They were Told about the Survey’s Content.” Box plots of participants’ post-survey change in affect according to their other vulnerability factors and what they were told about the survey’s content. Men’s results are shown in the top 4 panels, and women’s, in the bottom 4. Red dots indicate the mean change and black bars, the median change. Positive numbers indicate more sadness or tenseness post survey compared to pre-survey; negative numbers, less sadness or tenseness [file 12874_2022_1783_MOESM2_ESM.docx]

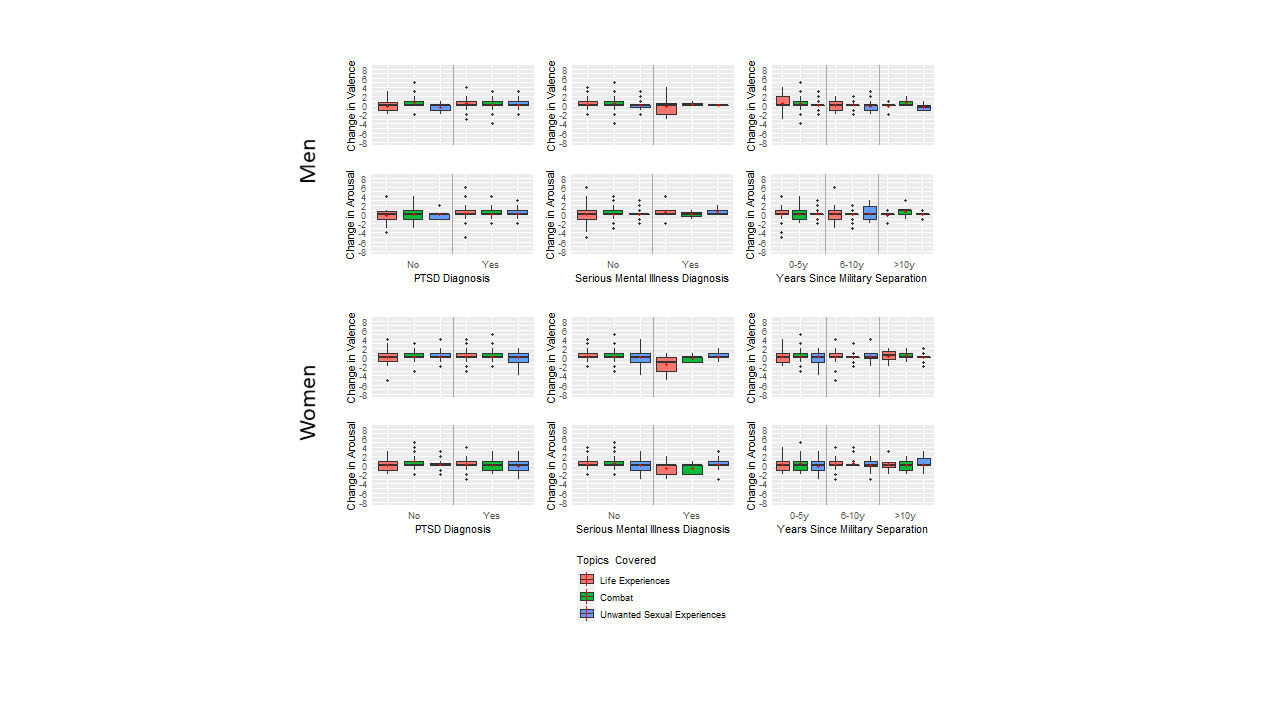
Supplementary Figure 1. “Post-Survey Change in Affect by Participants’ Other Vulnerability Factors and What They were Told about the Survey’s Content.” Box plots of participants’ post-survey change in affect according to their other vulnerability factors and what they were told about the survey’s content. Men’s results are shown in the top 4 panels, and women’s, in the bottom 4. Red dots indicate the mean change and black bars, the median change. Positive numbers indicate more sadness or tenseness post survey compared to pre-survey; negative numbers, less sadness or tenseness.
