# Supplementary material for: Impact of different cover letter information and incentives on Veterans’ emotional responses to an unsolicited mailed survey about military traumas: a randomized, 3x2x2 factorial trial
Source: BMC Med Res Methodol. 2022 Dec 1;22:308. doi: 10.1186/s12874-022-01783-7 (PMC9714177; doi:10.1186/s12874-022-01783-7)
Supplement: Supplementary file 4 — Additional file file 4: Supplementary Figure 3. “Post-Survey Change in Affect by Participants’ Military Trauma Exposures and the Incentive They were Promised.” Box plots of participants’ post-survey change in affect according to their military trauma history and the incentive they were promised. Men’s results are shown in the top 4 panels, and women’s, in the bottom 4. Red dots indicate the mean change and black bars, the median change. Positive numbers indicate more sadness or tenseness post survey compared to pre-survey; negative numbers, less sadness or tenseness. [file 12874_2022_1783_MOESM4_ESM.docx]

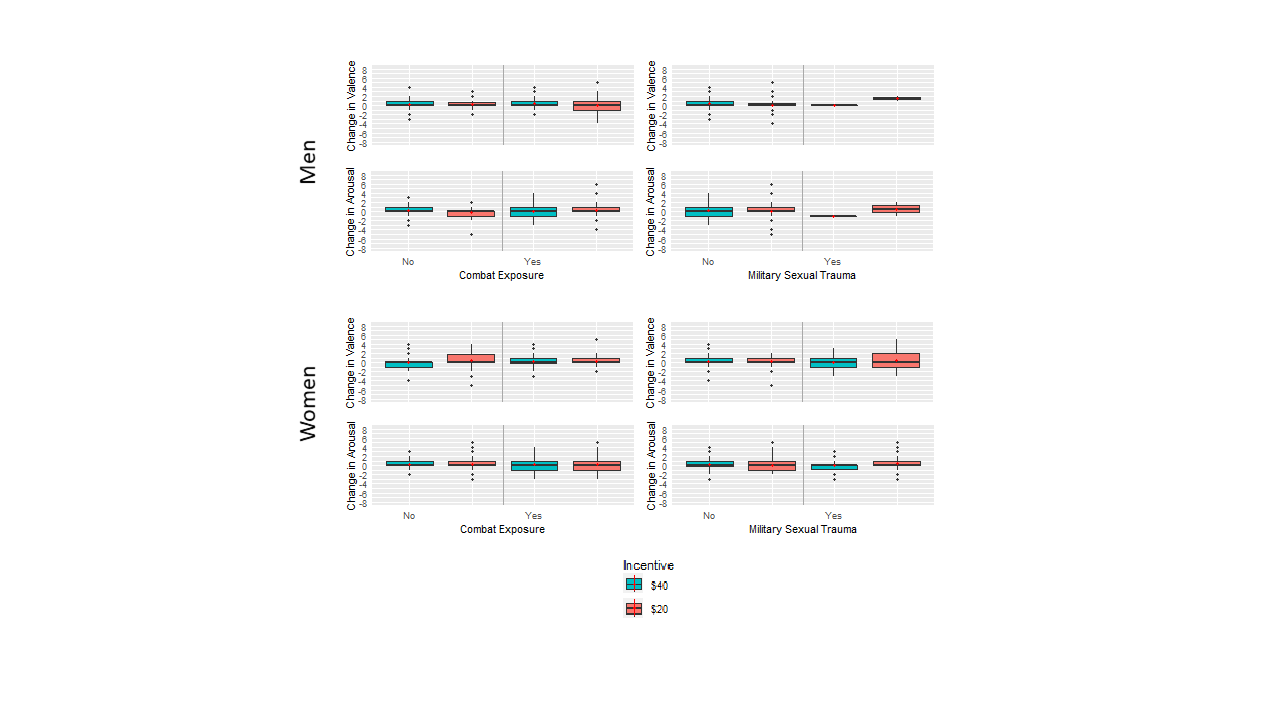


Supplementary Figure 3. “Post-Survey Change in Affect by Participants’ Military Trauma Exposures and the Incentive They were Promised.” Box plots of participants’ post-survey change in affect according to their military trauma history and the incentive they were promised. Men’s results are shown in the top 4 panels, and women’s, in the bottom 4. Red dots indicate the mean change and black bars, the median change. Positive numbers indicate more sadness or tenseness post survey compared to pre-survey; negative numbers, less sadness or tenseness.
