# Supplementary material for: Impact of different cover letter information and incentives on Veterans’ emotional responses to an unsolicited mailed survey about military traumas: a randomized, 3x2x2 factorial trial
Source: BMC Med Res Methodol. 2022 Dec 1;22:308. doi: 10.1186/s12874-022-01783-7 (PMC9714177; doi:10.1186/s12874-022-01783-7)
Supplement: Supplementary file 5 — Additional file 5: Supplementary Figure 4. “Post-Survey Change in Affect by Participants’ Other Vulnerability Factors and How their Name was Obtained.” Box plots of participants’ post-survey change in affect according to their other vulnerability factors and what they were told about how their name was obtained for inclusion in the study. Men’s results are shown in the top 4 panels, and women’s, in the bottom 4. Red dots indicate the mean change and black bars, the median change. Positive numbers indicate more sadness or tenseness post survey compared to pre-survey; negative numbers, less sadness or tenseness. [file 12874_2022_1783_MOESM5_ESM.docx]

**
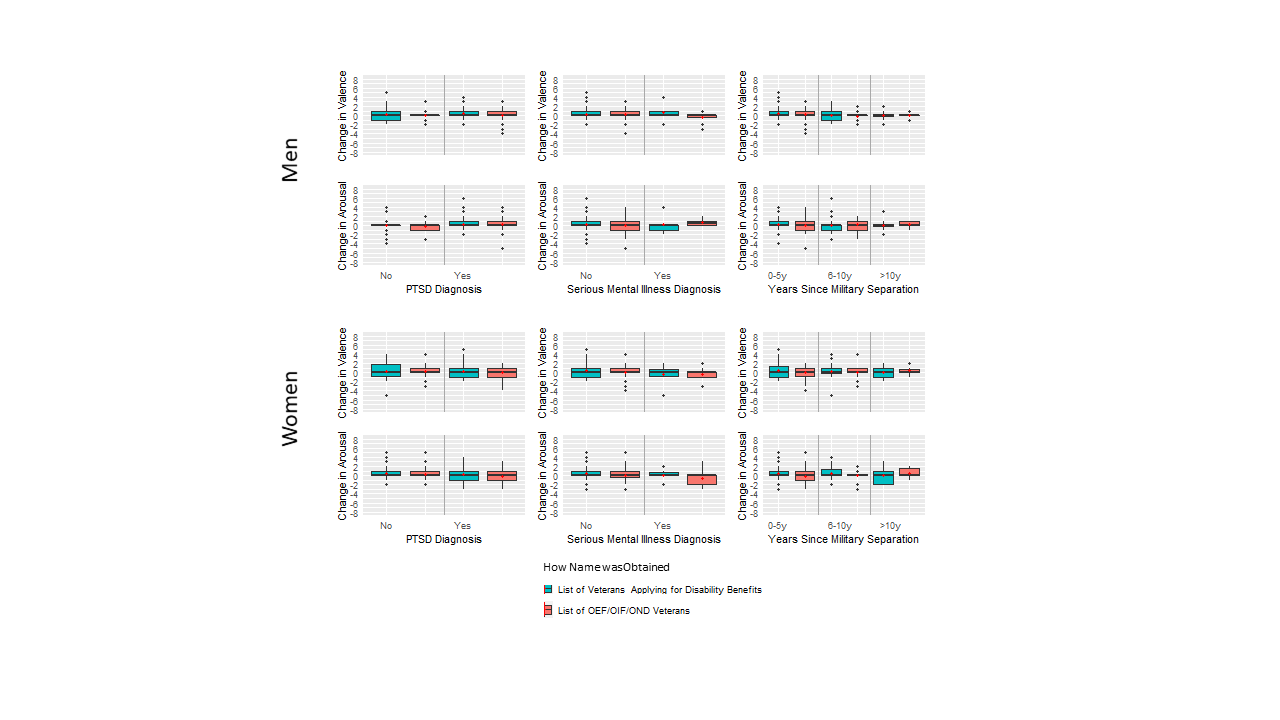
**

Supplementary Figure 4. “Post-Survey Change in Affect by Participants’ Other Vulnerability Factors and How their Name was Obtained.” Box plots of participants’ post-survey change in affect according to their other vulnerability factors and what they were told about how their name was obtained for inclusion in the study. Men’s results are shown in the top 4 panels, and women’s, in the bottom 4. Red dots indicate the mean change and black bars, the median change. Positive numbers indicate more sadness or tenseness post survey compared to pre-survey; negative numbers, less sadness or tenseness.
